# Instruments used to measure well-being, ill-being, and health-related lifestyle behaviors in students attending Italian universities: a systematic review

**DOI:** 10.3389/fpubh.2026.1787567

**Published:** 2026-04-20

**Authors:** Valentina Biscaldi, Jessica Guerini, Michela Ghelfi, Veronica Velasco

**Affiliations:** Department of Psychology, University of Milano-Bicocca, Milan, Italy

**Keywords:** Italian context, lifestyle behaviors, measurement instruments, mental health, risk and protective factors, systematic review, university students, well-being

## Abstract

**Background:**

The well-being of university students is increasingly recognized as a critical public health issue, influenced by complex interactions among psychological, behavioral, and contextual factors. Despite growing research, measurement tools often lack standardization and contextual specificity, limiting the understanding of students’ health. This review aimed to map and critically analyze instruments assessing well-being, ill-being, and health-related lifestyle behaviors among Italian university students, as well as the associated variables, including risk and protective factors.

**Methods:**

The systematic review followed PRISMA guidelines and included peer-reviewed studies published from 2010 onward, identified across five databases: Scopus, APA PsycInfo, PubMed, ERIC, and Web of Science. A structured data extraction process was applied to collect information on sample characteristics, health-related outcomes, and associated variables (protective and risk factors). Descriptive statistics were used to synthesize frequencies, proportions, and distributions of measurement instruments and constructs across the included studies.

**Results:**

A total of 223 studies were included. Samples were largely non-probabilistic and female-biased. Ill-being measures appeared exclusively in 66.3% of the studies, while 7.9% focused on well-being, and 25.8% included both. A total of 159 instruments assessing well-being and ill-being were identified. Of these, the majority measured ill-being (118 instruments), followed by instruments assessing well-being (28), and a smaller number addressing both constructs (13). In addition, 154 instruments measuring lifestyle were identified. Lifestyle behaviors were measured in a fragmented, health-risk-oriented manner, often lacking contextual influences. Individual predictors (130) were prioritized over relational and environmental factors (53). Few instruments were tailored specifically to university students, and many studies used non-validated or *ad hoc* tools, especially those developed during the COVID-19 pandemic.

**Discussion:**

Findings highlight the need for standardized, validated, and context-sensitive instruments to assess student health holistically.

## Introduction

University students represent a distinct population with specific developmental and life characteristics. Most students attend university while being in a specific stage of development called emerging adulthood (18–29 years). This period is marked by critical themes and transitional tasks, such as identity exploration, an increase in self-focus, instability, propensity to entertain possibilities, and feeling in-between adolescence and adulthood (1, 2). In addition, starting university itself represents a major life change, accompanied by important choices and shifts. Beginning an academic path implies having to decide about studies, adjusting to university demands, dealing with new learning methods, interacting with new people, rebuilding some parts of life, acquiring autonomy, eventually having a job, and, for some, moving away from home and distancing from usual support networks (3).

In this dynamic period of transition, characterized by developmental, academic, and social challenges, students can be more at risk of negative psychological effects (4). There are many reasons for this. First, vulnerability to mental illness reaches its peak between 18 and 25 years of age, and university students frequently fall within this group. Most disorders emerge in this age range, and three-quarters of those who have a mental illness developed their first symptoms during this life stage (5–8). Moreover, students’ mental health becomes strained when they start university. This strain tends to decrease over time, but it does not return to pre-university levels (9, 10). Results from the World Mental Health International College Student project by the World Health Organization (WHO) indicate that 35% of university students met criteria for at least one of the common lifetime disorders and 31% for one or more 12-month disorders. Major depressive disorder was the most frequent psychological problem (21.2% lifetime prevalence; 18.5% one-year prevalence), followed by generalized anxiety disorder (18.6–16.7%) (11). Depression in university students is higher than in the general population (271). In addition, during the recent COVID-19 pandemic, their mental condition has significantly declined, and some effects from that period could still be present (12, 13). Several factors specific to life during university could account for the worsening of students’ mental health: perceived academic pressure, financial or job-related struggles, adaptation to the new environment, social isolation, and lack of family or social support (14–16). It has also been estimated that during university years, the probability of disengaging from treatments is particularly high (5, 11).

The consideration of mental health problems in university students is important not only per se, but also because it influences other aspects of their lives and functioning. Mental issues during university can affect studying motivation and academic performance (5, 17, 18). In the long run, these conditions can predict college attrition and subsequent lower employment (5, 19). Moreover, mental disorders are a significant contributor to disability (20, 21). According to the WHO’s findings, 20.4% of university students present any severe role impairment: household-related tasks, academic work, close relationships, and social interactions. Among them, 42.9% have at least one psychological disorder. The ones associated with the highest probabilities of severe impairment were major depression, generalized anxiety disorder, and panic disorder (22).

Besides ill-being, university students’ well-being must be taken into consideration to account for their condition. Historically, theories, research, and practices have been dominated by a pathogenic perspective. In this framework, the term mental health has been used as a synonym for mental illness for a long time (23). In the last decades, a paradigm shift in favor of positive aspects, strengths, and resources has been advocated, and since then, studies from this perspective have grown rapidly (24). Despite these advances in the subject, knowledge on mental health and well-being is still variegated and fragmented, deriving from multiple perspectives, different fields, and contexts (25). Despite this theoretical advancement, most studies on university students continue to focus solely on mental health problems, while research examining well-being remains relatively scarce. A recent bibliometric mapping has shown that the vast majority of articles on this topic, conducted from 1975 to 2020, concerned mental problems and challenges, while positive aspects were underrepresented. This indicates that pathogenic approaches in the assessment of university students’ conditions have been more widespread, while salutogenic perspectives have remained less prominent (25). In the academic setting, students’ well-being has been associated with relevant variables, such as educational goals, engagement, accomplishments, and attrition (17, 26).

In addition to mental aspects, lifestyle and health-related behaviors are also relevant to gaining an understanding of university students’ situation. Generally, during university years, parental oversight decreases, particularly for those who move away from their families, and students gain more responsibility regarding their self-management (3). For this reason, the university period represents a crucial phase in which health-related habits, such as nutrition, physical activity, sleep, tobacco consumption, and alcohol intake, are acquired and maintained. Risky behaviors concerning these aspects start in adolescence but can consolidate and get worse between 18 and 30 years of age (27). Those who develop unhealthy habits during their higher education years could carry them into adulthood, and this can increase the risk of developing non-communicable diseases, such as cardiovascular pathologies, diabetes, lung problems, and facing early illness onset (27–31). Moreover, since the advent of smartphones, the Internet has become exponentially widespread, accessible, and embedded in daily life. This has had an impact on individuals’ routines, work, communication, and learning process, and nowadays university students use it for a wide range of purposes, such as studying, accessing social networks, gaming, and online shopping (32). Despite the notable advancements brought by these new technologies, the excessive use of smartphones can lead to physical and mental health difficulties in university students’ lives. For example, it can impair sleep, cause headaches, reduce concentration and academic performance, and increase anxiety, stress, and depression (33, 34). For these reasons, internet and smartphone use cannot be overlooked in the investigation of university students’ health-related lifestyle habits. On the bright side, healthy habits have been linked to well-being, quality of life, satisfaction in affective relationships, resilience, and self-esteem in higher education students (35–37).

Overall, as outlined, these topics encompass a vast heterogeneity of constructs and conceptualizations, including multiple terms, definitions, and frameworks (25, 38). Accordingly, a wide variety of measures have been used for their assessment (38). Currently, an agreement on which domains and measures are relevant to understand students’ condition is lacking, and this contributes to the presence of a fragmentary and unstructured body of work (39, 40). Given the multi-dimensional and context-dependent nature of university students’ health and well-being, an ecological framework offers a comprehensive lens for organizing the determinants of these outcomes. According to ecological models [e.g., (41)], individual characteristics are embedded within broader relational, institutional, and societal contexts that jointly influence health and behavior. This perspective supports distinguishing between individual-level, relational, and broader contextual factors when mapping the constructs and measurement tools used in the literature.

To our knowledge, no systematic review has mapped the variables and the instruments used to assess these dimensions in Italian university students over the last 15 years. As far as we know, the only extant review on this in the Italian context has evaluated these aspects under the specific conditions of the COVID-19 pandemic (42). Moreover, based on our current information, no systematic review has outlined the variables that have been associated with these constructs in university students in Italy. A broad array of different variables and relationships has been detected in the literature, and their mapping could be useful to obtain a comprehensive understanding of extant associations and to develop empirically supported interventions and practices (43).

For this reason, the present review aims to systematically identify and map the dimensions and measures used to assess well-being, ill-being, and health-related lifestyle behaviors in university students in Italy. As a secondary aim, it investigates the constructs and tools used to measure related risk and protective factors. The scope is to provide a broad and comprehensive overview of the existing dimensions and instruments adopted in this research field in Italy.

This review was conducted as an initial step within the PRO-BEN project, Universities for Psychological Well-being: From Prevention to Intervention (UNIBEN-PI), funded by the Italian Ministry of University and Research (MUR).

## Methods

### Information sources and search strategy

This systematic review was carried out following the Preferred Reporting Items for Systematic Reviews and Meta-Analyses (PRISMA) guideline (272).

The article search was conducted on five academic databases, namely Scopus, APA PsycInfo, PubMed, Education Resources Information Center (ERIC), and Web of Science. Studies were included if published from 2010 up to the date of the search (November 2024), to detect a broad picture of the tools used since the widespread diffusion of smartphones among young people (273). The selection was limited to peer-reviewed articles written in English or Italian. Only scientific papers were included; therefore, editorials, letters, reports, and books were excluded, as well as grey literature. For Scopus and Web of Science, the filters “article” and “review” were flagged. Search strings were composed as follows: keywords for university, the word “student*,” terms for the constructs of interest (well-being, ill-being, health-related lifestyle behaviors), and keywords indicating measures (e.g., questionnaire*). We adjusted the syntax to fit the specificities of each database. Full search syntaxes are available in Supplementary material 1.

### Eligibility

The inclusion criteria choice was informed by the PICO framework. However, given the descriptive aim of the present review, PICO was used solely as a guiding structure for defining eligibility criteria rather than as a framework for formulating intervention-focused research questions or evaluating effectiveness. For this reason, the model was adapted to better tailor the exploratory nature of the objectives. Specifically, to ensure the coverage of the search process, no comparators were defined, and outcomes were broadly conceptualized to capture the range of constructs and measurement tools used to assess well-being, ill-being, and health-related lifestyle behaviors among university students in Italy. This is in line with literature about the adaptation of PICO in reviews (44, 45).

Population:

- University students as the main topic of the study
- The sample does not include pathological subpopulations
- Italy is the country or one of the countries in which the study was conducted

Intervention:

- Only observational studies were admitted. Intervention studies and reviews were excluded
- At least one part of the study had to be quantitative

Comparator: This study did not explore specific comparators.

Outcome:

- The outcome variables had to be about well-being, ill-being, and health-related lifestyle behaviors
- Studies regarding the psychometric evaluation or the translation of a measurement scale were excluded
- Studies concerning the efficacy of interventions were excluded

### Study selection, data extraction, and data analysis

The screening process was performed using the digital tool “Rayyan” (274).

The results from the different academic database searches were combined, and duplicates were eliminated. To ensure a consistent application of the eligibility criteria, two authors (VB and JG) independently examined the contributions. These authors first screened the articles by titles and abstracts. Additional records were identified through bibliographical references of selected papers. After these phases, the remaining articles were fully read. During all the selection steps, disagreements were resolved by discussion to find a consensus with a third reviewer (VV).

A structured data extraction procedure was conducted for each article included in the review. For each article, we collected information regarding the study and sample characteristics (size and representativeness of the sample, gender distribution and subpopulation, country and period of data collection), the instruments used to assess health-related outcomes, and the variables associated with those outcomes.

Health-related outcomes were grouped into three main categories: (1) psychological well-being and ill-being, (2) lifestyle-related behaviors, and (3) perceived health status. Determinants were organized into four main domains: (1) sociodemographic characteristics, (2) individual factors, (3) relational or contextual factors, and (4) COVID-19-specific variables.

Descriptive analyses were performed to determine the presence and diversity of measurement instruments across the reviewed literature. We calculated the number of distinct instruments used for each outcome and associated variable, the percentage of validated instruments, and the proportion of studies that included at least one tool. In addition, we identified instruments specifically developed or adapted for use in the university student population. To ensure a consistent classification and reliable calculations, this process was performed by two authors (JG and MG) independently. In the event of inconsistencies, the results were discussed until agreement was reached.

### Risk of bias and methodological quality

The aim of this review was to map the dimensions and measurement instruments used to assess well-being, ill-being, and health-related lifestyle behaviors, as well as related risk and protective factors, among university students in Italy. Given this descriptive focus on measurement practices, a full risk of bias assessment was not undertaken.

However, to provide a methodological context for the interpretation of the findings, we conducted a simplified quality appraisal based on selected criteria adapted from JBI Critical Appraisal Checklist for Analytical Cross-Sectional Studies (46). This appraisal focused on methodological characteristics relevant to the use and reporting of measurement instruments rather than on internal validity or causal inference.

Specifically, the following aspects were examined: study design (cross-sectional vs. longitudinal); sample representativeness (probabilistic/random sampling, non-probabilistic sampling with population reference information, or no information reported); reporting of basic sample characteristics (at least one among gender, age, or course of study); use of at least one validated measurement scale for outcomes (well-being, ill-being, or lifestyle-related behaviors) or for risk and protective factors; and type of statistical analyses conducted (descriptive only vs. descriptive and inferential).

With regard to study design, the majority of the included studies adopted a cross-sectional design (*N =* 204; 91.5%) and a minority employed longitudinal approaches (*N =* 19; 8.5%).

Only a limited number of studies reported information on sample representativeness. Specifically, 1.8% of the included studies employed probabilistic or randomized sampling methods (*N =* 4), while 10.8% used non-probabilistic samples but provided population reference information (*N =* 24). The vast majority of studies (87.4%; *N =* 195) did not report sufficient information to assess sample representativeness.

All included studies (*N =* 223) reported at least one basic sample characteristic, such as gender, age, or course of study.

Regarding measurement quality, most studies employed at least one validated instrument to assess either outcomes or risk and protective factors. Specifically, 87.4% of the studies (*N =* 195) used at least one validated scale, whereas 12.6% (*N =* 28) did not use validated instruments for all measured constructs. Among these latter studies, the majority focused on lifestyle-related outcomes (*N =* 26), while only two assessed well-being or ill-being.

Finally, with respect to data analysis, 98.2% of studies (*N =* 219) conducted inferential analyses in addition to descriptive statistics, whereas only 1.8% (*N =* 4) relied exclusively on descriptive analyses.

Overall, these findings highlight important methodological limitations, particularly regarding sample representativeness and the predominance of cross-sectional designs. Nevertheless, the majority of studies adhered to the other methodological criteria, which should be kept in mind when interpreting the distribution and use of instruments identified in this review.

Full references for each study corresponding to the methodological quality are provided in Supplementary material 2.

## Results

**Figure 1:**
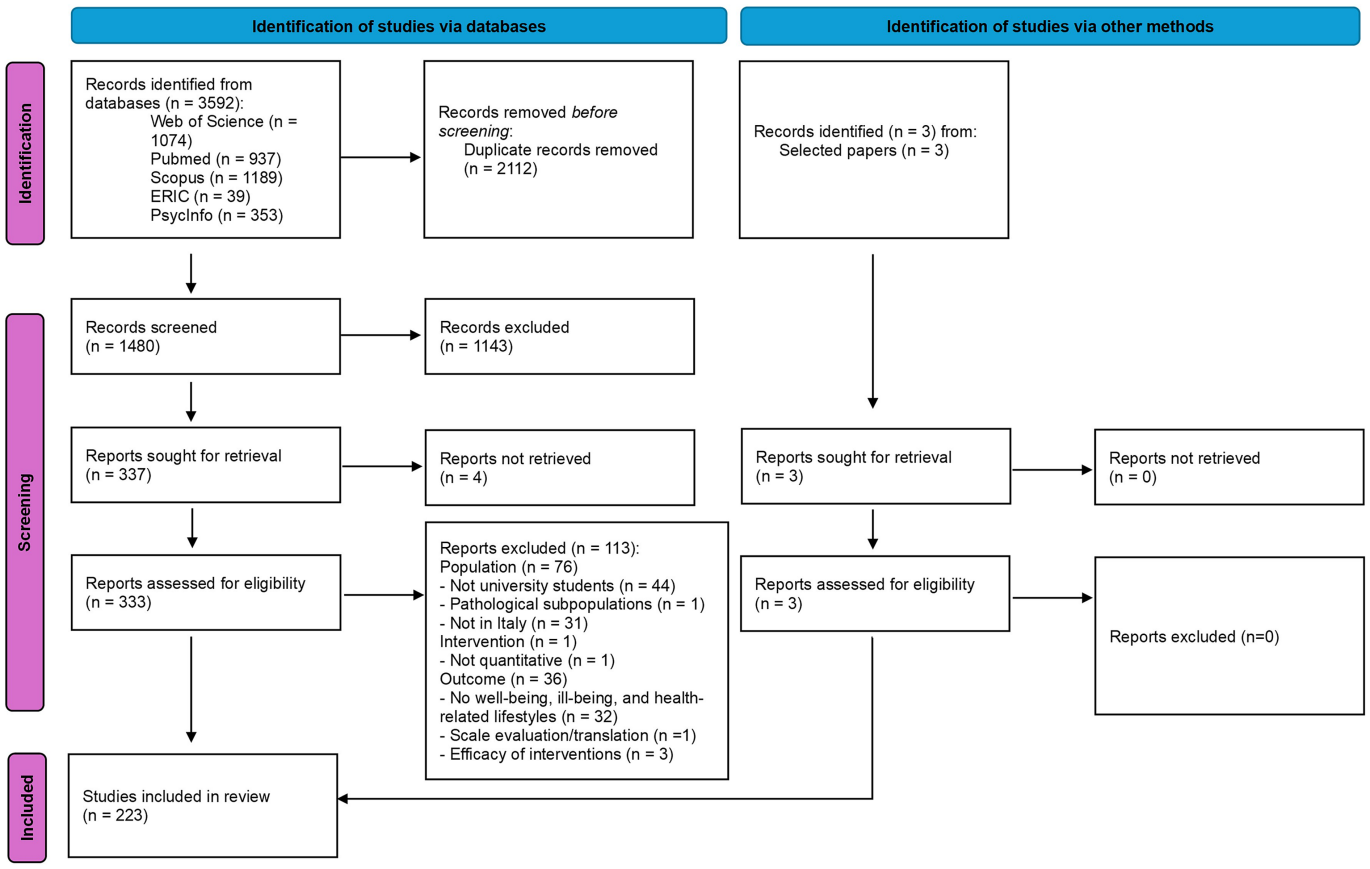
PRISMA flowchart of the literature search and screening process.

Figure 1 shows the PRISMA flow diagram of the selection process, which led to the inclusion of 223 papers in the review. The full list of included studies is provided in Table 1.

**Table 1:** List of studies included in the literature review with the corresponding citation (authors and year of publication).

No.	Author, year	No.	Author, year	No.	Author, year	No.	Author, year
1	Aiello et al., 2022 (54)	28	Bonaccorso et al., 2024 (187)	55	Chiodo et al., 2023 (100)	82	Dell’Osso et al., 2019 (231)
2	Alessandri et al., 2017 (59)	29	Bottesi et al., 2012 (275)	56	Cicchella et al., 2022 (105)	83	Dell’Osso et al., 2022 (236)
3	Aliberti et al., 2019 (64)	30	Brytek-Matera et al., 2017 (196)	57	Ciocca et al., 2018 (110)	84	Delvecchio et al., 2023 (241)
4	Allen et al., 2023 (69)	31	Buizza et al., 2022 (201)	58	Cipolletta et al., 2021 (115)	85	Di Consiglio et al., 2021 (244)
5	Angelillo et al. (74)	32	Buizza et al., 2022 (206)	59	Colombo and Cellini, 2024 (120)	86	Di Fabio and Kenny, 2018 (249)
6	Angelone et al., 2023 (80)	33	Buizza et al., 2023 (211)	60	Colonnello et al., 2022 (125)	87	Di Fabio and Kenny, 2018 (254)
7	Aristovnik et al., 2021 (85)	34	Bulfone et al., 2023 (216)	61	Colonnello et al., 2023 (76)	88	Di Fabio et al., 2022 (259)
8	Armando et al., 2010 (89)	35	Cabras et al., 2023 (221)	62	Commodari et al., 2021 (134)	89	Di Giacomo et al., 2021 (264)
9	Backhaus et al., 2020 (94)	36	Campo et al., 2022 (226)	63	Comotti et al., 2024 (139)	90	Di Martino et al., 2024 (268)
10	Backhaus et al., 2020 (99)	37	Canale et al., 2016 (230)	64	Comparcini et al., 2022 (144)	91	Eskin et al., 2016 (56)
11	Bagordo et al., 2013 (104)	38	Canzan et al., 2019 (235)	65	Concerto et al., 2022 (149)	92	Eskin et al., 2019 (61)
12	Bassanini et al., 2024 (109)	39	Capone, 2018 (240)	66	Coniglio, 2012 (154)	93	Esposito et al., 2020 (66)
13	Bassi et al., 2023 (114)	40	Capone et al., 2020 (17)	67	Conte et al., 2023 (158)	94	Esposito et al., 2024 (71)
14	Bastiani et al., 2019 (119)	41	Caricati and Ferrari, 2021 (248)	68	Cosentino et al., 2024 (163)	95	Falgares et al., 2018 (77)
15	Belingheri et al., 2019 (124)	42	Carollo et al., 2024 (253)	69	Craig et al., 2023 (168)	96	Ferrante et al., 2013 (82)
16	Belingheri et al., 2020 (129)	43	Carpi et al., 2022 (258)	70	Curcio et al., 2022 (173)	97	Ferrara et al., 2022 (87)
17	Bellini et al., 2022 (133)	44	Carpi and Vestri, 2023 (263)	71	Curcio et al., 2024 (178)	98	Ferrari et al., 2017 (91)
18	Bert et al., 2022 (138)	45	Carpita et al., 2024 (267)	72	D’Egidio et al., 2020 (183)	99	Ficarra et al., 2022 (96)
19	Bertani et al., 2020 (143)	46	Casale and Fioravanti, 2015 (55)	73	Dakanalis et al., 2016 (188)	100	Fino et al., 2021 (101)
20	Bertocchi et al., 2021 (148)	47	Casali et al., 2024 (60)	74	De Micheli et al., 2021 (192)	101	Fiori Nastro et al., 2024 (106)
21	Biasi et al., 2017 (153)	48	Castelli et al., 2023 (65)	75	De Pasquale et al., 2018 (197)	102	Franzoi et al., 2024 (111)
22	Biasi et al., 2018 (157)	49	Cavallo et al., 2023 (70)	76	De Pasquale et al., 2021 (202)	103	Gafforelli et al., 2020 (116)
23	Bimonte et al., 2020 (162)	50	Cecilia et al., 2016 (75)	77	De Pasquale et al., 2021 (207)	104	Gallè et al., 2019 (121)
24	Biondi et al., 2021 (167)	51	Cena et al., 2021 (81)	78	De Vincenzo and Carpi, 2024 (212)	105	Gallè et al., 2023 (126)
25	Biraghi and Tortorano, 2010 (172)	52	Cerutti et al., 2022 (86)	79	Dell’Osso et al., 2014 (217)	106	Gallè et al., 2025 (130)
26	Biscaldi et al., 2023 (177)	53	Cerutti et al., 2023 (90)	80	Dell’Osso et al., 2015 (222)	107	Galvin et al., 2022 (135)
27	Bo et al., 2014 (182)	54	Chayinska and Mari, 2014 (95)	81	Dell’Osso et al., 2018 (227)	108	Generali et al., 2021 (140)

No.	Author, year	No.	Author, year	No.	Author, year	No.	Author, year
109	Ghislieri et al., 2023 (145)	138	Luciano et al., 2021 (67)	167	Poscia et al., 2015 (209)	196	Saulle et al., 2013 (132)
110	Ghislieri et al., 2023 (150)	139	Lupi et al., 2015 (72)	168	Prigitano et al., 2020 (214)	197	Scandroglio et al., 2023 (137)
111	Giampaoli et al., 2021 (155)	140	Lyrakos, 2012 (78)	169	Procentese et al., 2020 (219)	198	Schettino et al., 2022 (142)
112	Gianfredi et al., 2017 (159)	141	Majori et al., 2017 (83)	170	Protano et al., 2023 (224)	199	Schramlova et al., 2024 (147)
113	Gianfredi et al., 2018 (164)	142	Majori et al., 2017 (83)	171	Provenzano et al., 2018 (229)	200	Scuri et al., 2019 (152)
114	Giangrasso et al., 2022 (169)	143	Manuli et al., 2022 (92)	172	Provenzano et al., 2019 (233)	201	Servidio et al., 2022 (156)
115	Gorrasi et al., 2020 (174)	144	Maran and Begotti, 2021 (97)	173	Provenzano et al., 2019 (238)	202	Servidio et al., 2024 (161)
116	Gramaglia et al., 2019 (179)	145	Martinucci et al., 2018 (102)	174	Provenzano et al., 2020 (242)	203	Sette et al., 2023 (166)
117	Guglielmetti et al., 2022 (184)	146	Marzilli et al., 2020 (107)	175	Provenzano et al., 2020 (246)	204	Somma et al., 2020 (171)
118	Guidotti et al., 2022 (189)	147	Mascia et al., 2023 (112)	176	Provenzano et al., 2020 (251)	205	Stefa-Missagli et al., 2025 (176)
119	Guidotti et al., 2024 (193)	148	Mastrokoukou et al., 2024 (117)	177	Quintiliani et al., 2022 (256)	206	Tancredi et al., 2022 (181)
120	Guidotti et al., 2024 (198)	149	Matranga et al., 2020 (122)	178	Raiola and Aliberti, 2021 (261)	207	Teleman et al., 2015 (186)
121	Ingusci et al., 2023 (203)	150	Meda et al., 2023 (127)	179	Rania et al., 2014 (266)	208	Tettamanti et al., 2016 (191)
122	La Fauci et al., 2023 (208)	151	Mereu et al., 2021 (131)	180	Riccobono et al., 2020 (270)	209	Tinella et al., 2022 (195)
123	La Rosa and Commodari, 2023 (213)	152	Messina et al., 2016 (136)	181	Rinella et al., 2018 (58)	210	Tonetti et al., 2012 (200)
124	Laganà et al., 2021 (218)	153	Messina et al., 2021 (141)	182	Riva Crugnola et al., 2021 (63)	211	Tugnoli et al., 2022 (205)
125	Leombruni et al., 2021 (223)	154	Monaci et al., 2013 (146)	183	Rodakowska et al., 2020 (68)	212	Uras et al., 2012 (210)
126	Levante et al., 2024 (228)	155	Moscatelli et al., 2023 (151)	184	Roggio et al., 2021 (73)	213	Uras et al., 2012 (215)
127	Licata et al., 2024 (232)	156	Moscatelli et al., 2023 (30)	185	Romano et al., 2019 (79)	214	Vallone et al., 2023 (220)
128	Licata et al., 2024 (237)	157	Mucci et al., 2014 (160)	186	Rometsch et al., 2024 (84)	215	Vigiani et al., 2024 (225)
129	Licata et al., 2024 (27)	158	Nola et al., 2023 (165)	187	Romito et al., 2019 (88)	216	Villani et al., 2021 (18)
130	Lietz et al., 2018 (245)	159	Orsolini et al., 2022 (170)	188	Santangelo et al., 2018 (93)	217	Villano et al., 2021 (234)
131	Litwic-Kaminska et al., 2023 (250)	160	Paulo et al., 2024 (175)	189	Santangelo et al., 2018 (98)	218	Viselli et al., 2021 (239)
132	Lo Moro et al., 2021 (255)	161	Perilli et al., 2021 (180)	190	Santangelo et al., 2018 (103)	219	Višnjić et al., 2018 (243)
134	Lo Moro et al., 2023 (260)	162	Pighi et al., 2018 (185)	191	Santangelo et al., 2019 (108)	220	Vitale et al., 2020 (247)
133	Lodi et al., 2022 (265)	163	Piumatti et al., 2016 (190)	192	Santangelo et al., 2019 (113)	221	Vitiello et al., 2016 (252)
135	Lombardo et al., 2014 (269)	164	Piumatti, 2018 (194)	193	Santangelo et al., 2020 (118)	222	Zurlo et al., 2021 (257)
136	Lonati et al., 2024 (57)	165	Polese et al., 2025 (199)	194	Santangelo et al., 2022 (123)	223	Zurlo et al., 2022 (262)
137	Luca et al., 2013 (62)	166	Porru et al., 2021 (204)	195	Santangelo et al., 2022 (128)		

## Characteristics of included studies

The characteristics of the included studies are summarized in Table 2. Information reported includes sample size, gender distribution, participants’ field of study, country, and data collection period. This overview provides a comprehensive understanding of the populations and contexts investigated across the reviewed literature. Full references for each study corresponding to these characteristics are provided in Supplementary material 3.

**Table 2:** Sample and data characteristics of the papers included in the review (*N =* 223).

Paper characteristics	*N* (%)
Sample size	
N < 500	102 (45.7%)
501 < N < 1,000	60 (26.9%)
N > 1,000	61 (27.4%)
Gender distribution	
Balanced	76 (34.1%)
Female prevalence (over 65%)	128 (57.4%)
Male prevalence (over 65%)	5 (2.2%)
Missing	14 (6.3%)
Students’ field of study	
Specific areas	73 (32.8%)
Various areas	75 (33.6%)
Missing	75 (33.6%)
Country	
Italy	199 (89,2%)
Italy + others	24 (10.8%)
Data collection period	
Pre-COVID-19	109 (48.9%)
During COVID-19 (March 2020–December 2022)	80 (35.9%)
Post-COVID-19	12 (5.4%)
Missing	22 (9.8%)

Nearly half of the studies involved relatively small samples, including fewer than 500 participants. In terms of gender distribution, female participants were predominant in most cases. The majority of research was conducted exclusively within Italy, while only about 10% of studies were multinational.

It is also worth noting that, although numerous studies had already been conducted before the COVID-19 pandemic, there was a marked surge of interest in the mental health and well-being of university students during the pandemic period.

## Objective 1: identification of constructs and instruments used to assess health outcomes (well-being, ill-being, and lifestyle) in university students

### Psychological well-being and ill-being

Table 3 provides a quantitative summary of the instruments used, categorized by outcome type (well-being, ill-being, or both). The table includes the number of articles in which at least one instrument was applied, the total number of tools of each outcome, the proportion of validated instruments, the number of distinct tools after merging different versions of the same scale (e.g., short versions), and the subset of instruments specifically developed or adapted for the university student population.

**Table 3:** Summary of instruments for psychological well-being and ill-being.

Instrument type	Total articles	Total instruments		% validated instruments	Unique instruments (merged)^a^	University context instruments^b^
		*N*	%			
Negative-oriented scales	150	118	74.2%	86.4%	107	14
Problematic state related to lifestyle^c^	52	33		84.9%		
Positive-oriented scales	38	13	8.2%	87.6%	28	8
Mixed-oriented scales	28	28	17.6%	84.6%	13	0
Overall	177	159	100%	85.9%	148	22

^a^Unique instruments (merged) refers to instruments measuring the same construct that were grouped together despite minor adaptations or different versions used across studies. ^b^University-context instruments include tools specifically developed for or validated in university student populations. ^c^These measurement scales are included in the calculation of Negative-oriented scales.

Across the 223 articles included in the review, a total of 159 distinct instruments were used to assess psychological well-being and ill-being (148 when different versions of the same instrument were merged).

The reviewed literature reveals a marked predominance of papers aimed at assessing psychological distress and ill-being, while articles focusing specifically on positive well-being are relatively scarce. Of the 223 reviewed articles, 178 assessed outcomes related to well-being and/or ill-being. Among these, 66.3% included measures of ill-being exclusively, whereas a smaller proportion focused exclusively on well-being (7.9%) or on both well-being and ill-being (25.8%).

Similarly, when considering the distinct instruments identified, the majority targeted ill-being (74.2%), while 17.6% assessed well-being and 8.2% addressed both. The majority of scales were oriented toward negative psychological states, including depression, anxiety, stress, and burnout. Some instruments provided a general assessment of psychological discomfort, while others focused on specific symptom dimensions or clinical markers.

Among the instruments used to assess ill-being, several focused directly on problematic health behaviors. These included tools measuring substance abuse and dependence, disordered eating, and sleep disorders. Such instruments were designed to detect maladaptive health-related behaviors as expressions of psychological ill-being.

Positive-oriented scales primarily address constructs such as life satisfaction, flourishing, and general quality of life, but their representation across studies is limited compared to measures of distress. Furthermore, only a few studies employed instruments that consider both positive and negative aspects of mental health within the same framework.

Most of the instruments (>80%) used across the studies were validated, thus representing a good level of accuracy in measuring health outcomes.

Overall, just 13.8% of the 159 total instruments were specifically tailored to the university student population. Among these instruments specifically designed for the university context, 14 focused on ill-being, 8 on well-being, and none assessed both.

Table 4 includes a list of the most frequently employed instruments, categorized by the nature of the construct assessed and sorted decreasingly by frequency of use. The table reports names of the instruments, the number of articles in which each tool appears, and the number of items for each instrument.

**Table 4:** Most frequently used instruments for psychological well-being and ill-being.

Instrument type	Construct measured	Instrument name	*N* items	Frequency (*N* articles)
Negative-oriented scales	General	SCL-90-R Symptom Checklist-90-Revised	90	14
		DASS-21 Depression, Anxiety, and Stress Scale - DASS-42	21–42	13
		HADS Hospital Anxiety and Depression Scale	14	4
		ASR Adult Self Report DSM-oriented	120	4
	Stress Burnout	PSS-10 Perceived Stress Scale	10	11
	Depression	BDI Beck Depression Inventory	21	17
		PHQ-9 Patient Health Questionnaire	9	6
	Anxiety	STAI State Trait Anxiety Inventory	40	7
		BAI Beck Anxiety inventory	21	7
		GAD-7 Generalized Anxiety Disorder scale	7	6
	Overall			89
*Negative-oriented lifestyle scales	Eating disorder	ORTO-15 - ORTO-R	15–6	10
		EAT-26 Eating Attitude Test	26	7
	Sleep disorder	ISI Insomnia Severity Index	7	4
	Alcohol use disorder	AUDIT-C Alcohol Use Disorders Identification Test-Consumption	3	11
	Internet addiction	IAT Internet Addiction Test	20	5
	Overall			37
Positive-oriented scales	General	SWLS Satisfaction With Life Scale	5	8
		MHC-SF Mental Health Continuum Short Form	14	5
		FS Flourishing Scale	8	4
		MLM Meaningful Life Measure	23	3
	Overall			20
Mixed-oriented scales	General	GHQ-12 General Health Questionnaire-12	12	12
		PANAS Positive and Negative Affect Schedule	20	6
	Overall			18

^a^Only instruments used in at least three studies are reported.

### Lifestyles

Table 5 summarizes, for each lifestyle domain—physical health habits, substance-related behaviors, and leisure and social behaviors—and their respective subdomains, the number of articles in which at least one tool was used, the number of instruments identified, and the percentage of validated instruments.

**Table 5:** Summary of instruments used to assess lifestyle-related behaviors.

Lifestyle domain	Subdomain	Total articles	Total instruments		% validated instruments
			*N*	%	
Physical health habits	Sleep	22	10		50%
	Physical activity	40	27		14.8%
	Eating habits	45	31		32.3%
	Overall	65	68	44.2%	32.4%
Substance-related behaviors	Alcohol use	22	15		13.3%
	Tobacco use	46	19		15.8%
	Cannabis and other drug use	7	7		0%
	Supplements/medications use	17	20		0%
	Overall	62	61	39.6%	7.3%
Leisure and social behaviors	Media use	9	12		15.4%
	Leisure time	5	5		0%
	Sexuality and reproduction	6	8		0%
	Overall	21	25	16.2%	5.2%
Lifestyle total		106	154	100%	15%

Several included papers investigated university students’ lifestyles. Among the 223 included articles, 106 assessed at least one lifestyle factor (47.5% of the total sample). The reviewed studies revealed a greater emphasis on physical health habits (e.g., sleep quality, physical activity, and eating habits), which were the most frequently assessed behaviors (48% of the 223 articles). The second most investigated category was consumption-related behaviors (alcohol consumption, cannabis and other drug use, tobacco use, and intake of supplements or medications), which were investigated in 41.3% of the papers. To a much lesser extent, leisure-related activities were analyzed in 9% papers; this category included media use, leisure activities, and sexuality, which tend to be less systematically evaluated. A similar pattern emerged when considering the total number of lifestyles’ instruments identified (154 different instruments): 44.2% measured physical health behaviors, 39.6% targeted consumption-related behaviors, and only 16.2% focused on leisure activities. Most of the instruments identified in the reviewed studies focused on the measurement of a single lifestyle domain. Only two tools were designed to assess multiple lifestyle behaviors in an integrated manner. Moreover, no instruments were found that specifically addressed lifestyle habits in the context of university student life.

Lifestyle-related behaviors were generally assessed through separate indicators, rather than through comprehensive or multidimensional tools. In the majority of cases, data on lifestyle were collected using *ad hoc* questionnaires, self-constructed items, or adaptations of existing tools, rather than through standardized or validated instruments. Only a very limited number of validated tools (14.2%) were used across the studies, indicating a lack of consistency and comparability in the assessment of student lifestyle factors.

Table 6 summarizes the most frequently used instruments for each lifestyle-related behavior, reporting the number of articles in which each tool appears and the number of items for each instrument.

**Table 6:** Most frequently used instruments for each lifestyle domain.

Lifestyle domain	Subdomain	Instrument name	*N* items	Frequency (*N* articles)
Physical behaviors	Sleep	PSQI Pittsburgh Sleep Quality Index	19	13
	Physical activity	Physical activity (yes/no)	1	18
		IPAQ International Physical Activity Questionnaire	27	9
	Eating habits	Type of diet followed (e.g., mediterranean/vegan/vegetarian/omnivorous diet)	1	11
		FFQ Food Frequency Questionnaire	72	3
	Overall			54
Substance-related behaviors	Alcohol use	Alcohol consumption (yes/no)	1	8
		Frequency of alcohol consumption (regular, occasional, never)	1	8
	Tobacco use	Current tobacco use (yes/no)	1	20
		Tobacco-related condition (smoker, quitter, or non-smoker)	1	9
	Cannabis and other drug use	Experienced drugs in lifetime (yes/no)	1	4
	Supplements/medication use	Dietary supplements use (yes/no)	1	4
	Overall			53
Leisure and social behaviors	Media use	Number of hours usually spent using electronic devices during the day	1	3
	Leisure time	Leisure activities (pub, cultural activities, disco, sports)	1	3
	Sexuality and reproduction	Sexual behaviors (yes/no)	1	4
	Overall			10

^a^Only instruments used in at least three studies are reported.

### Perceived health status

Perceived overall health represents a global and subjective assessment of one’s physical and mental condition. Despite its relevance as a general indicator of health status, this outcome is measured sporadically within the reviewed literature. Of the 223 papers reviewed, only 8.5% included at least one instrument addressing this dimension.

A total of seven different tools were identified, none of which were validated. In most cases, health perception was assessed using a single-item question, typically formulated as “How would you perceive your overall health?”, with response options such as low, medium, or high. Only one study employed the standardized and WHO-recommended version of this item: “How is your health in general?”.

This limited and non-standardized use of tools suggests that, although global health perception could serve as a useful integrative measure of well-being, it remains an underutilized and inconsistently assessed construct in research on university students.

## Objective 2: Identification of Constructs and Instruments Used to Assess Variables Associated with Health Outcomes (Risk and Protective Factors) in University Students

Consistent with the ecological framework outlined in the Introduction, the variables associated with outcomes are presented according to the levels used in this review: socio-demographic characteristics, individual-level variables, and relational and contextual factors. In addition, we considered COVID-19-specific factors, which represent historical and contextual influences characteristic of recent years. This organization facilitates a structured overview of the evidence and highlights the multi-level nature of determinants of well-being and lifestyle.

### Socio-demographic characteristics

Socio-demographic variables were extensively utilized across the reviewed literature. In fact, 71.7% of studies included sociodemographic variables. The instruments reported have been categorized into several key domains.

The Biological and Personal domain encompasses characteristics such as gender (present in 56.5% of the articles), age (40.4%), and body mass index (BMI) (13.9%). Less frequently assessed variables in this category include country of origin and nationality, each reported in 2.7% of the studies.

The Residential Status domain includes variables related to living arrangements, such as living with family, residing away from home, or on−/off-campus housing, which were reported in 9.9% of the studies. More specific aspects, including living conditions, cohabitation status, and family environment, were considered in 3.1% of the studies, while regional or local residence was assessed in 4.0%. Within the Economic and Financial domain, perceived economic status (categorized as low, medium, or high) was reported in 8.1% of the articles.

The Family Context domain often included variables such as parents’ educational levels or qualifications, which were measured in 6.3% of the studies.

Regarding the Relationship Status domain, marital or relationship status was reported in 10.3% of the studies, while sexual orientation was mentioned in 2.2%.

Some socio-demographic variables were less commonly investigated, including Medical History and Conditions, Religious Beliefs, and a few general or unspecified socio-demographic factors, with only one article including the latter.

Finally, specific university-related variables were also examined, highlighting the contextual importance of academic factors. This included degree course or type (7.2%), year of study (13.9%), academic discipline or field (7.6%), university country (5.4%), and whether students were working alongside their studies (6.3%).

Aggregated data on sociodemographic variables are presented in Table 7. The table reports the number of studies in which at least one variable was measured, the total number of instruments used, and the percentage of validated instruments.

**Table 7:** Instruments used to assess variables associated with health outcomes.

Type of variables	Sub-variables	Construct	Total articles	Total instruments		% validated instruments
				*N*	%	
Socio-Demographic Characteristics			160	53	18.1%	/
Individual-Level Variables			100	130	44.4%	63.9%
	Personality traits and characteristics	Traits	23	16		93.7%
		Attachment style	4	2		100%
		Personality	17	9		8.9%
		Overall	40	27		92.6%
	Personal resources and skills	Self-efficacy	3	3		100%
		Coping	11	9		100%
		Resilience	4	3		100%
		Self-esteem	5	4		100%
		Health literacy	1	1		100%
		Health and climate knowledge and beliefs	3	6		0%
		Various resources	14	14		85.7%
		Overall	35	41		78%
	University-specific individual variables	Resources and strategies	11	13		84.6%
		Academic performance and study-related behaviors	23	20		45%
		Beliefs	6	10		10%
		Overall	32	43		48.8%
	Health conditions and vulnerabilities	Overall	13	11		0%
	Future perspectives	Overall	7	8		100%
Relational and contextual variables			34	53	18.1%	45.6%
	General Life Context	General	7	10		50%
		Family	8	6		66.7%
		Social capital	2	2		100%
		Social support	4	5		80%
		Stigma	1	1		100%
		Overall	23	24		66.7%
	Psychological and Medical Support	Overall	10	19		0%
	University-Specific Context	Peers, professors, academic relationships, and climate	6	8		62.5%
		Learning context	2	2		100%
		Overall	9	10		70%
COVID-19-specific factors			31	57	19.4%	17.5%

### Individual-level variables

Individual-level variables represent a broad and multifaceted category of predictors, encompassing a range of personal characteristics, resources, vulnerabilities, and experiences. In this review, these variables were organized into five main subdomains: (1) personality traits and characteristics, (2) personal resources and skills, (3) university-specific individual variables, (4) health conditions and vulnerabilities, and (5) future perspectives.

The data showed that Personality Traits and Characteristics appeared most frequently, being reported in 17.9% of the studies, followed by Personal Resources and Skills, which were measured in 15.7% of the papers. University-Specific Individual Variables were assessed in 14.3% of the studies, while Health Conditions and Vulnerabilities were examined in 5.8%. Lastly, Future Perspectives were explored in only 3.1% of the studies.

Regarding the total number of distinct instruments identified (*N =* 130), 20.8% measured Personality Traits, 31.5% focused on Personal Resources and Skills, and 33% assessed University-Specific Individual Variables. In contrast, only 8.5% measured Health Conditions, and just 6.2% addressed Future Perspectives.

The first group referred to stable or semi-stable individual characteristics such as personality traits, attachment styles, and, in some cases, personality disorders. These traits often serve as underlying predispositions that can either increase vulnerability or offer protection in the face of academic and life stressors. Notably, 92.6% of the instruments used to assess these characteristics were validated.

The second category focused on personal resources and adaptive skills, including constructs such as self-efficacy, coping strategies, resilience, self-esteem, health literacy, and various other resources, and knowledge and beliefs about health and climate. These were often conceptualized as individual strengths but can also reflect a person’s capacity to interact effectively with their environment. Importantly, 78% of the instruments used to measure these constructs were validated.

The third group included university-specific individual variables, with domains such as academic self-regulation, study-related skills and behaviors, performance perceptions, and personal beliefs related to academic life. Of the instruments used to assess these variables, 48.8% were validated.

The fourth category included both physical and psychological/psychiatric conditions that may influence students’ well-being. Among the most frequently reported are somatic symptoms with psychological components, such as headaches, gastrointestinal problems, and musculoskeletal issues, alongside clinical or subclinical psychological disorders. In this category, no single instrument emerged as predominant, and the tools used were often single, non-validated items.

Finally, future perspectives—especially those related to academic and occupational outcomes— were explored both in their protective (e.g., ambition, motivation) and risk-oriented (e.g., anxiety, uncertainty) dimensions. Although no single instrument emerged as dominant across studies, all instruments identified within this domain were validated, indicating a limited yet heterogeneous assessment of this area.

The data on individual variables, including the specific sub-variables and their respective constructs, are presented in Table 7.

### Relational and contextual variables

Relational and contextual variables refer to the broader social environments in which students are embedded and the quality of the relationships they maintain. In the included studies, these variables were operationalized in general life context factors through constructs such as family relationships, social support, social capital, and stigma; university-specific aspects including relationships with peers and professors, perceptions of academic climate, and learning context; and demand for psychological and medical support.

A limited number of instruments were identified to assess these constructs. Variables related to the general life context, such as family relationships, social support, and social capital, were measured in relatively few studies (10.3%), often employing heterogeneous tools. University-specific relational dimensions, such as interactions with peers and faculty members or perceptions of the academic environment, were addressed in a small subset of studies (4%), with limited standardization of measurement tools. Finally, variables concerning the demand for psychological and medical support, including help-seeking behaviors, use of counseling services, and access to psychiatric care, were explored in approximately 4.5% of the studies. In most cases, these variables were assessed using simple, non-standardized measures, frequently based on single dichotomous items (e.g., yes/no questions).

Regarding the total number of distinct instruments identified (*N =* 53), 45.3% measured general life context variables, of which 66.7% were validated instruments, 35.8% assessed psychological and medical support variables, none of which were validated, and 18.9% measured university-specific relational dimensions, with 70% of these instruments being validated.

Overall, the number of studies and tools identified in this category was noticeably lower compared to those targeting individual-level variables. The data related to relational and contextual variables, including constructs and sub-variables, are presented in Table 7.

### COVID-19-specific factors

In total, 13.9% of the studies investigated constructs specifically related to the COVID-19 context within university settings. These variables were diverse and clustered into five main domains: behaviors (e.g., protective actions, health habits), beliefs and knowledge (e.g., perceived severity, misinformation), active involvement (e.g., academic engagement during lockdowns), distress or psychological discomfort (e.g., anxiety, fear, uncertainty), and university-specific stressors (e.g., stress related to online learning or institutional changes).

Across these studies, 57 different measurement tools were used. However, the vast majority consisted of single-item or *ad hoc* instruments with limited psychometric validation. Only 17.5% of these instruments were identified as previously validated or psychometrically tested.

This heterogeneity in measurement approaches reflects both the novelty of the context and the urgency with which researchers sought to capture rapidly evolving experiences during the health crisis. However, it also highlights the need for more standardized and validated tools to ensure comparability and methodological rigor in future studies.

The aggregated data related to COVID-19-specific factors are presented in Table 7.

### Exploratory analyses: temporal trends and study scope

As an additional set of exploratory analyses, temporal trends were first examined across the pre-COVID, during-COVID, and post-COVID periods. For this analysis, 158 articles have been included, considering those studies that assessed outcomes related to well-being and/or ill-being and the period of data collection could be classified according to the COVID-19 pandemic timeline. Pre-COVID studies measured exclusively negative outcomes in 69.1% of cases, both positive and negative outcomes in 20.2%, and only positive outcomes in 10.7%. During COVID, 58.5% focused on negative outcomes, 35.4% on both, and 6.1% on positive outcomes exclusively. Notably, post-COVID studies assessed exclusively negative outcomes (100%), with only one study employing instruments assessing relational and contextual factors. Overall, a stable trend was observed over time, with studies consistently emphasizing negative outcomes over positive or mixed outcomes and primarily focusing on individual-level factors rather than relational or contextual factors. Given the limited number of post-COVID studies (*N =* 12, compared with 109 pre-COVID and 80 during COVID), these trends should be interpreted with caution.

Additionally, the 24 multinational studies were examined. Instruments measuring negative outcomes and individual-level factors predominated, while instruments assessing positive or mixed outcomes and relational or contextual factors were used less frequently. Among the multinational studies, 60% used instruments measuring only negative outcomes (ill-being), 10% used instruments measuring only positive outcomes (well-being), and 30% used instruments measuring both positive and negative outcomes. COVID-specific instruments appeared during the pandemic but were rare (*N =* 2). In total, the studies employed 24 different instruments measuring negative outcomes, 5 measuring positive outcomes, and 3 measuring mixed outcomes, highlighting the use of diverse instruments across studies, with several studies using multiple instruments to assess different dimensions of well-being and ill-being. Across these studies, almost all outcome instruments were validated (22 of 24), with the two exceptions assessing lifestyle outcomes rather than well-being.

Taken together, these exploratory analyses indicate stable and consistent measurement practices across time and study scope, highlighting the predominant focus on instruments measuring negative outcomes and individual-level factors in the literature on university students’ well-being.

## Discussion

The present review aims to identify the dimensions and measures used to assess well-being, ill-being, and health-related lifestyle behaviors in university students in Italy. As a secondary aim, it investigates the constructs and tools used to measure related risk and protective factors.

The contribution lies in mapping the wide range of variables and associations identified in the literature, which often appear heterogeneous and uncoordinated. This synthesis may support a more integrated understanding of existing evidence and inform the development of empirically grounded interventions and practices.

### Fragmentation and dispersion of measures

The overall picture that emerged from this review is one of significant heterogeneity and fragmentation in measurement practices. A wide variety of constructs have been assessed using a large number of tools, many of which are non-validated, developed *ad hoc*, or adapted without formal testing.

This dispersion of instruments, as noted by Dodd et al. (38), poses several challenges. It limits comparability across studies, hampers the accumulation of evidence, and complicates meta-analytical efforts. Moreover, the use of unvalidated or poorly documented measures reduces the reliability and validity of findings, increasing the risk of methodological bias.

These findings suggest a need for greater standardization in research on student health, possibly through collaborative initiatives aimed at identifying core outcome sets or shared measurement frameworks. Establishing a shared set of core instruments would facilitate more meaningful cross-study comparisons and strengthen the relevance of research findings for informing health and education policy. Within the “PRO-BEN” project, a recent initiative on university student well-being funded by the Italian Ministry of University and Research (MUR), for instance, there is an opportunity to promote harmonization of tools across studies and institutions, fostering cumulative knowledge and more consistent practices.

Importantly, the heterogeneity observed in the use of multiple instruments to assess similar outcomes is not unique to Italy. Exploratory analyses of 24 multinational studies revealed a comparable pattern of measurement fragmentation, confirming that this is an international phenomenon. However, 22 of these studies used validated instruments to assess well-being, ill-being, or lifestyle outcomes, suggesting a higher methodological rigor in outcome assessment at the international level compared to the Italian context.

### Predominance of a pathogenic approach and limited holistic assessment

One of the most salient findings of this review aligns with international evidence suggesting that research on university students’ mental health is still largely dominated by a pathogenic paradigm (23, 25). The majority of the studies included in the present review focused on psychological distress, particularly depression, anxiety, and stress, using validated instruments originally developed for general population samples. Conversely, tools assessing positive aspects of mental health, such as life satisfaction, flourishing, and overall psychological well-being, were significantly less frequent.

Furthermore, very few instruments simultaneously measured both well-being and ill-being, highlighting a persisting conceptual divide between positive and negative mental health dimensions. This gap mirrors the historical conflation of mental health with mental illness, as previously critiqued in the literature (23, 24) and underscores the underrepresentation of salutogenic approaches in student mental health research. These findings align with the UK scoping review by Dodd et al. (38), which reported inconsistencies in defining and measuring university student well-being and a predominant focus on subjective experiences. They also mirror the broader international literature’s emphasis on pathogenic approaches to mental health (25), supporting the international generalizability of our results and the need for more integrative approaches assessing both well-being and ill-being.

In quantitative terms, among the 223 reviewed articles, 178 assessed outcomes related to well-being and/or ill-being. Among these, 66.3% included measures exclusively of ill-being, whereas smaller proportions focused exclusively on well-being (7.9%) or on both well-being and ill-being (25.8%). This pattern is mirrored in the measurement tools themselves: 74.2% of the instruments identified were designed to assess ill-being, 17.6% targeted well-being, and only 8.2% addressed both dimensions.

Importantly, the predominant focus on ill-being observed in Italian studies reflects a broader international pattern. Exploratory analyses of the 24 multinational studies included in this review showed a similar trend.

Finally, it is worth noting that constructs such as perceived health status were assessed only sporadically and often through non-validated instruments. This highlights a persisting difficulty in capturing student health comprehensively and holistically, reflecting limitations in the conceptualization of overall health in current research. These gaps further underscore the need for future studies to develop and adopt systematic, validated tools for assessing students’ health in a comprehensive manner.

### Lifestyle behaviors: a fragmented, health-oriented vision

Lifestyle behaviors were another core focus of the review, yet the findings revealed a fragmented approach. This fragmentation appears to be largely due to the wide variety of instruments employed to measure lifestyle factors, including many *ad hoc* tools. To enhance greater homogeneity, future research could benefit from adopting tools from internationally recognized monitoring surveys.

Moreover, the literature appears to prioritize behaviors with a direct or perceived link to ill-being or health risk, such as alcohol and substance abuse, sleep disorders, disordered eating, and excessive smartphone use. These behaviors are often framed within a health-risk paradigm, mirroring medical or clinical concerns more than lifestyle patterns shaped by social, psychological, or environmental influences (27, 31).

This narrow and pathology-driven lens can limit our understanding of lifestyle as a dynamic and contextual aspect of students’ everyday lives. For example, dimensions such as leisure activities, sexual health, social relationships, and digital media use are either overlooked or measured through non-standardized, ad hoc items (17, 25, 26).

### Imbalance between individual and contextual predictors

In the analysis of predictors associated with health outcomes, the review found a significant imbalance between individual-level and relational or contextual-level factors. Among the studies reviewed, more than 70% focused on individual-level predictors, such as personality traits, emotional regulation, coping skills, or psychological vulnerabilities, whereas only 25% included relational or contextual variables, such as family support, peer relationships, university climate, or academic environment. Notably, exploratory analyses of multinational studies included in this review revealed a similar pattern: instruments measuring individual-level factors predominated, while relational/contextual factors were less frequently assessed, suggesting that this imbalance is consistent beyond the Italian context.

While individual factors are undoubtedly important, an overreliance on them may lead to an excessive emphasis on personal responsibility and a neglect of structural and contextual influences on student health. While acknowledging the relevance of individual aspects, it is crucial to also embrace broader social, relational, and contextual dimensions from an interdisciplinary perspective (47). Moreover, relational and contextual variables were often measured with fewer tools, frequently lacking standardization, and were generally framed in a secondary or supporting role.

This pattern reflects a broader tendency in research to privilege individual and intrapsychic factors over ecological and environmental variables (41, 48). However, studies have shown that relational and institutional factors, including students’ sense of belonging, perceived support from faculty, and engagement with peers, play a critical role in shaping academic and psychological outcomes (15–17, 26).

Notably, only a small number of studies explored students’ help-seeking behavior or use of mental health services, despite evidence indicating that university students frequently underutilize available support services (49). This suggests a missed opportunity to better understand and address barriers to care.

This asymmetry suggests the need for a more ecological and integrative perspective that acknowledges the role of the broader university environment in shaping health outcomes, recognizing the reciprocal relationship between the organizational context and the health, well-being, and performance of students (50). Considering contextual factors is essential not only for theoretical comprehensiveness but also for the design of multi-level interventions targeting both individual vulnerabilities and environmental stressors.

### Lack of university-specific measurement tools

Another key observation is the limited availability and use of measurement tools that are specific to the university student population. Most instruments used across the reviewed studies (86.2%) were developed for the general population, without reference to the unique characteristics of student life. Only 13.8% of the tools were explicitly tailored to the university context.

Importantly, no instruments specifically addressed lifestyle within the university context, missing a crucial opportunity to examine how academic, social, and developmental transitions shape students’ habits and routines. This is particularly relevant considering that the university period represents a critical developmental window during which long-term health-related habits are formed and stabilized (28, 29).

This lack of context-specific measurement is particularly problematic considering the transitional and developmentally unique phase of emerging adulthood (1) and the specific challenges that characterize university life, such as academic demands, relocation, autonomy, identity exploration, and changes in social support systems (3). Generic tools may fail to capture these aspects, potentially reducing both the sensitivity and ecological validity of assessments.

In the Italian context, this gap may be further exacerbated by limited national efforts toward instrument development and validation within academic populations.

Developing and validating university-specific tools could contribute to a more accurate and relevant assessment of student well-being, enabling researchers and practitioners to identify needs more precisely and design more contextually appropriate interventions. This aligns with the Health Promoting Universities framework, which emphasizes the importance of creating supportive environments and systemic approaches tailored to the unique context of each institution to effectively enhance health outcomes (51).

### Influence of the COVID-19 pandemic: a hybrid dimension

The COVID-19 pandemic represented a pivotal moment in drawing attention to student mental health. During this period, students’ psychological well-being significantly declined, and some of the negative effects may persist today (12, 13).

A notable number of studies conducted after 2020 included COVID-19-specific variables, highlighting both individual vulnerabilities and environmental disruptions. These variables, such as perceived impact of the pandemic, social isolation, online learning difficulties, and fear of contagion, do not fit neatly into traditional categories, as they simultaneously reflect internal psychological states and external contextual conditions. Importantly, exploratory analyses of multinational studies indicated that COVID-specific factors were rarely assessed internationally, with only 2 studies including such measures, suggesting that this intense focus on pandemic-related outcomes was largely an Italian research phenomenon.

Interestingly, most of these variables were assessed through *ad hoc* measures (47 studies), including single items or non-validated scales, while only a minority (10 studies) employed validated instruments. This again highlights the proliferation of non-standardized tools in the field and the urgent need for validated instruments capable of capturing complex, cross-cutting experiences.

The pandemic underscored the relevance of understanding student well-being not solely as an individual attribute, but as a dynamic construct shaped by broader social and environmental conditions (52). It also exposed structural gaps in how we conceptualize and measure student health, further emphasizing the need for integrative, flexible, and context-sensitive assessment tools (53).

### Sample characteristics: limited representativeness and gender imbalance

An important limitation emerging from this review concerned the characteristics of the samples used in the included studies. Most of the studies were conducted on non-probabilistic, convenience samples recruited from single institutions, often within specific courses or faculties. This poses significant concerns regarding the generalizability of the findings, as such samples may not adequately reflect the diversity of the broader university student population.

Moreover, a strong gender imbalance was observed across studies, with a predominant representation of female participants. This overrepresentation may be due to higher response rates among women in mental health research, yet it limits the ability to draw conclusions applicable to male or gender-diverse students, whose experiences and vulnerabilities may differ significantly.

Other potentially relevant subgroups, such as international students, working students, students with disabilities, or those from underrepresented socio-economic backgrounds, were rarely analyzed or even identified. This underreporting further restricts the inclusiveness of current research and may obscure the needs of vulnerable or marginalized populations.

Future studies should prioritize more representative and stratified sampling strategies and report socio-demographic variables more consistently. This would enhance the external validity of findings and support the development of equity-informed interventions that address the needs of all students, not just the most accessible ones.

### Limitations

This systematic review presents several limitations that should be acknowledged. First, the review only included studies conducted in the Italian context, which limits the generalizability of the findings to other cultural or educational settings. The Italian context has specific features, such as variations in student age, university organization, longer family co-residence, and a more gradual transition to economic and residential autonomy. Nevertheless, as already discussed, the patterns observed largely align with international trends, confirming the results of previous studies (38), suggesting that the findings may be relevant beyond Italy. This choice reflects the aim of focusing on tools used in the Italian context, as the breadth of international literature would have made a detailed analysis of instruments unfeasible. Second, grey literature, such as unpublished studies, conference proceedings, or institutional reports, was excluded, potentially omitting relevant data and contributing to publication bias. Third, the quality of the studies included was not formally assessed. However, as our focus was on the instruments and constructs investigated rather than the study outcomes, a quality check was obtained directly on the measurement tools by calculating the proportion of validated instruments. Additionally, the review was restricted to studies published from 2010 onwards; while this ensures contemporary relevance, it may have excluded earlier foundational work on student well-being and lifestyle. Finally, the role of perceived health status was not examined in depth due to its sporadic and non-standardized measurement in the included studies. This gap highlights the need for future research to focus on perceived health status using validated, standardized tools, in order to more accurately capture students’ subjective health perceptions and their connection to lifestyle behaviors and well-being. These limitations highlight the need for future reviews to adopt broader inclusion criteria and integrate methodological quality assessments to enhance the comprehensiveness and reliability of the evidence base.

## Conclusion

This systematic review provides a comprehensive overview of the instruments used to assess well-being, ill-being, and health-related lifestyle behaviors, as well as associated variables such as protective and risk factors, among university students in Italy over the past 15 years. The findings reveal several critical patterns and gaps in the current literature, with implications for both research and practice. One promising opportunity to address these gaps is offered by the recent “PROBEN” project, funded by the Italian Ministry of University and Research (MUR), which aims to promote student well-being. Within this initiative, there is potential to facilitate the harmonization of tools across studies and institutions, fostering cumulative knowledge and more consistent practices.

## Data Availability

The original contributions presented in the study are included in the article/Supplementary material, further inquiries can be directed to the corresponding author.
