# Supplementary material for: Instruments used to measure well-being, ill-being, and health-related lifestyle behaviors in students attending Italian universities: a systematic review
Source: Front Public Health. 2026 Apr 20;14:1787567. doi: 10.3389/fpubh.2026.1787567 (PMC13136261; doi:10.3389/fpubh.2026.1787567)
Supplement: Supplementary file 1 [file Supplementary_File_1.docx]

**SUPPLEMENTARY MATERIALS**

**SUPPLEMENTARY MATERIAL 1**

PsycInfo Query Strings

(abstract(universit*) OR abstract(colleg*) OR abstract(academ*) OR abstract(bachelor*) OR abstract(master*) OR abstract(undergrad*) OR abstract(under-grad*) OR abstract(“higher education”) OR abstract(“post-secondary”) OR abstract(postsecondary) OR abstract(“tertiary education”)) AND abstract(student*) AND (abstract(wellbeing) OR abstract(“well-being”) OR abstract(“quality of life”) OR abstract(happ*) OR abstract(“life satisfaction”) OR abstract(“satisfaction with life”) OR abstract(wellness) OR abstract(health*) OR abstract(hedon*) OR abstract(eudaimon*) OR abstract(good life) OR abstract(anxi*) OR abstract(depress*) OR abstract(burnout) OR abstract(disorder*) OR abstract(stress*) OR abstract(distress*) OR abstract(symptom*)) AND (abstract(questionnaire*) OR abstract(index*) OR abstract(scale*)) AND allfields(Ital*)

Web of Science Query String

(AB=(universit*) OR AB=(colleg*) OR AB=(academ*) OR AB=(bachelor*) OR AB=(master*) OR AB=(undergrad*) OR AB=(under-grad*) OR AB=(“higher education”) OR AB=(post-secondary) OR AB=(postsecondary) OR AB=(“tertiary education”)) AND AB=(student*) AND (AB=(wellbeing) OR AB=(well-being) OR AB=(“quality of life”) OR AB=(happ*) OR AB=(“life satisfaction”) OR AB=(“satisfaction with life”) OR AB=(wellness) OR AB=(health*) OR AB=(hedon*) OR AB=(eudaimon*) OR AB=(“good life”) OR AB=(anx*) OR AB=(depress*) OR AB=(burnout) OR AB=(disorder*) OR AB=(stress*) OR AB=(distress*) OR AB=(symptom*)) AND (AB=(questionnaire*) OR AB=(scale*) OR AB=(index*)) AND ALL=(Ital*)

Scopus Query String

(ABS(universit*) OR ABS(colleg*) OR ABS(academ*) OR ABS(bachelor*) OR ABS(master*) OR ABS(undergrad*) OR ABS(under-grad*) OR ABS(“higher education”) OR ABS(post-secondary) OR ABS(postsecondary) OR ABS(“tertiary education”)) AND ABS(student*) AND (ABS(wellbeing) OR ABS(well-being) OR ABS(“quality of life”) OR ABS(happ*) OR ABS(“life satisfaction”) OR ABS(“satisfaction with life”) OR ABS(wellness) OR ABS(health*) OR ABS(hedon*) OR ABS(eudaimon*) OR ABS(“good life”) OR ABS(anx*) OR ABS(depress*) OR ABS(burnout) OR ABS(disorder*) OR ABS(stress*) OR ABS(distress*) OR ABS(symptom*)) AND (ABS(questionnaire*) OR ABS(scale*) OR ABS(index*)) AND ALL(Ital*)

PubMed Query String

(universit*[Title/Abstract] OR colleg*[Title/Abstract] OR academ*[Title/Abstract] OR bachelor*[Title/Abstract] OR master*[Title/Abstract] OR undergrad*[Title/Abstract] OR under-grad*[Title/Abstract] OR "higher education"[Title/Abstract] OR "post-secondary"[Title/Abstract] OR postsecondary[Title/Abstract] OR "tertiary education"[Title/Abstract]) AND (student*[Title/Abstract]) AND (wellbeing[Title/Abstract] OR "well-being"[Title/Abstract] OR "quality of life"[Title/Abstract] OR happ*[Title/Abstract] OR "life satisfaction"[Title/Abstract] OR "satisfaction with life"[Title/Abstract] OR wellness[Title/Abstract] OR health*[Title/Abstract] OR hedon*[Title/Abstract] OR eudaimon*[Title/Abstract] OR good life[Title/Abstract] OR anxi*[Title/Abstract] OR depress*[Title/Abstract] OR burnout[Title/Abstract] OR disorder*[Title/Abstract] OR stress*[Title/Abstract] OR distress*[Title/Abstract] OR symptom*[Title/Abstract]) AND (questionnaire*[Title/Abstract] OR index*[Title/Abstract] OR scale*[Title/Abstract]) AND (Ital*)

ERIC Query String via ProQuest (Education Resources Information Center)

(abstract(universit*) OR abstract(colleg*) OR abstract(academ*) OR abstract(bachelor*) OR abstract(master*) OR abstract(undergrad*) OR abstract(under-grad*) OR abstract("higher education") OR abstract("post-secondary") OR abstract(postsecondary) OR abstract("tertiary education")) AND abstract(student*) AND (abstract(wellbeing) OR abstract("well-being") OR abstract("quality of life") OR abstract(happ*) OR abstract("life satisfaction") OR abstract("satisfaction with life") OR abstract(wellness) OR abstract(health*) OR abstract(hedon*) OR abstract(eudaimon*) OR abstract("good life") OR abstract(anxi*) OR abstract(depress*) OR abstract(burnout) OR abstract(disorder*) OR abstract(stress*) OR abstract(distress*) OR abstract(symptom*)) AND (abstract(questionnaire*) OR abstract(index*) OR abstract(scale*)) AND allfields(Ital*)

**SUPPLEMENTARY MATERIAL 2**

Methodological characteristics of the studies included in the review with the respective reference number (N=223)

| **Methodological quality indicators** | **N° papers (%)** | **References (n° paper)** |
| --- | --- | --- |
| *Study design* | | |
| Cross-sectional | 204 (91.5%) | 1, 3, 4, 5, 6, 7, 8, 9, 10, 11, 13, 14, 15, 16, 17, 18, 19, 20, 21, 22, 23, 24, 25, 26, 27, 28, 29, 30, 31, 32, 35, 36, 37, 38, 40, 41, 42, 43, 44, 45, 46, 47, 48, 49, 50, 51, 53, 54, 55, 56, 57, 58, 59, 60, 61, 62, 63, 64, 65, 66, 67, 68, 69, 70, 71, 72, 74, 75, 76, 77, 78, 79, 80, 81, 82, 83, 84, 86, 87, 88, 89, 90, 91, 92, 95, 96, 97, 99, 101, 102, 103, 104, 105, 106, 107, 108, 109, 110, 111, 112, 113, 114, 115, 116, 117, 118, 119, 120, 121, 122, 124, 125, 126, 127, 128, 129, 130, 131, 132, 133, 134, 135, 136, 137, 138, 139, 141, 142, 143, 144, 145, 146, 147, 148, 149, 150, 151, 152, 153, 154, 155, 156, 157, 158, 159, 160, 161, 162, 163, 165, 166, 167, 168, 169, 170, 171, 172, 173, 174, 175, 176, 177, 178, 179, 180, 181, 182, 183, 184, 185, 186, 187, 188, 189, 190, 191, 192, 193, 194, 195, 196, 197, 199, 200, 201, 202, 205, 206, 207, 208, 209, 210, 211, 212, 213, 214, 215, 216, 217, 219, 220, 221, 222, 223 |
| Longitudinal | 19 (8.5%) | 2, 12, 33, 34, 39, 52, 73, 85, 93, 94, 98, 100, 123, 140, 164, 198, 203, 204, 218 |
| *Representativeness* | | |
| Probabilistic/random sample | 4 (1.8%) | 128, 148, 175, 215 |
| Non-probabilistic sample with population reference information | 24 (10.8%) | 7, 23, 32, 35, 36, 60, ,71, 74, 89, 94, 97, 112, 121,126, 136, 141, 147, 159, 160, 175, 194, 195, 212, 213, 221 |
| No information on representativeness / Non-representative sample | 195 (87.4%) | 1, 2, 3, 4, 5, 6, 8, 9, 10, 11, 12, 13, 14, 15, 16, 17, 18, 19, 20, 21, 22, 24, 25, 26, 27, 28, 29, 30, 31, 33, 34, 37, 38, 39, 40, 41, 42, 43, 44, 45, 46, 47, 48, 49, 50, 51, 52, 53, 54, 55, 56, 57, 58, 59, 61, 62, 63, 64, 65, 66, 67, 68, 69, 70, 72, 73, 75, 76, 77, 78, 79, 80, 81, 82, 83, 84, 85, 86, 87, 88, 90, 91, 92, 93, 95, 96, 98, 99, 100, 101, 102, 103, 104, 105, 106, 107, 108, 109, 110, 111, 113, 114, 115, 116, 117, 118, 119, 120, 122, 123, 124, 125, 127, 129, 130, 131, 132, 133, 134, 135, 137, 138, 139, 140, 142, 143, 144, 145, 146, 149, 150, 151, 152, 153, 154, 155, 156, 157, 158, 161, 162, 163, 164, 165, 166, 167, 168, 169, 170, 171, 172, 173, 174, 176, 177, 178, 179, 180, 181, 182, 183, 184, 185, 186, 187, 188, 189, 190, 191, 192, 193, 196, 197, 198, 199, 200, 201, 202, 203, 204, 205, 206, 207, 208, 209, 210, 211, 214, 216, 217, 218, 219, 220, 222, 223 |
| *Sample characteristics reported* | | |
| At least one among gender, age, or course of study | 223 (100%) | 1, 2, 3, 4, 5, 6, 7, 8, 9, 10, 11, 12, 13, 14, 15, 16, 17, 18, 19, 20, 21, 22, 23, 24, 25, 26, 27, 28, 29, 30, 31, 32, 33, 34, 35, 36, 37, 38, 39, 40, 41, 42, 43, 44, 45, 46, 47, 48, 49, 50, 51, 52, 53, 54, 55, 56, 57, 58, 59, 60, 61, 62, 63, 64, 65, 66, 67, 68, 69, 70, 71, 72, 73, 74, 75, 76, 77, 78, 79, 80, 81, 82, 83, 84, 85, 86, 87, 88, 89, 90, 91, 92, 93, 94, 95, 96, 97, 98, 99, 100, 101, 102, 103, 104, 105, 106, 107, 108, 109, 110, 111, 112, 113, 114, 115, 116, 117, 118, 119, 120, 121, 122, 123, 124, 125, 126, 127, 128, 129, 130, 131, 132, 133, 134, 135, 136, 137, 138, 139, 140, 141, 142, 143, 144, 145, 146, 147, 148, 149, 150, 151, 152, 153, 154, 155, 156, 157, 158, 159, 160, 161, 162, 163, 164, 165, 166, 167, 168, 169, 170, 171, 172, 173, 174, 175, 176, 177, 178, 179, 180, 181, 182, 183, 184, 185, 186, 187, 188, 189, 190, 191, 192, 193, 194, 195, 196, 197, 198, 199, 200, 201, 202, 203, 204, 205, 206, 207, 208, 209, 210, 211, 212, 213, 214, 215, 216, 217, 218, 219, 220, 221, 222, 223 |
| Not reported | 0 |  |
| *Validated measurement for outcomes or for risk and protective factors* | | |
| At least one validated instrument | 195 (87.4%) | 1, 2, 3, 4, 5, 6, 8, 9, 10, 11, 12, 13, 14, 16, 17, 18, 19, 20, 21, 22, 24, 26, 27, 28, 29, 30, 31, 32, 33, 34, 35, 36, 37, 39, 40, 41, 42, 43, 44, 45, 46, 47, 48, 49, 51, 52, 53, 54, 55, 57, 58, 59, 60, 61, 62, 63, 64, 65, 66, 67, 68, 69, 70, 71, 72, 73, 74, 75, 76, 77, 78, 79, 80, 81, 82, 83, 84, 85, 86, 87, 88, 89, 90, 91, 92, 93, 94, 95, 96, 97, 98, 99, 100, 101, 102, 103, 106, 107, 108, 109, 110, 111, 113, 114, 115, 116, 117, 118, 119, 120, 121, 122, 123, 124, 125, 126, 127, 128, 129, 130, 131, 132, 133, 134, 135, 137, 138, 139, 140, 143, 144, 146, 147, 148, 149, 150, 151, 152, 153, 154, 156, 157, 159, 160, 161, 163, 164, 165, 166, 167, 169, 172, 173, 174, 176, 177, 179, 180, 181, 182, 183, 184, 185, 186, 187, 188, 189, 190, 192, 194, 195, 196, 197, 198, 199, 201, 202, 203, 204, 205, 206, 209, 210, 211, 212, 213, 214, 215, 216, 217, 218, 219, 220, 222, 223 |
| Only non-validated instruments | 28 (12.6%) | 7, 15, 23, 25, 38, 50, 56, 104, 105, 112, 136, 141, 142, 145, 155, 158, 162, 168, 170, 171, 175, 178, 191, 193, 200, 207, 208, 221 |
| *Statistical analysis* | | |
| Inferential analyses | 219 (98.2%) | 1, 2, 3, 4, 5, 6, 7, 8, 9, 10, 11, 12, 13, 14, 15, 16, 17, 18, 19, 21, 22, 23, 24, 25, 26, 27, 28, 30, 31, 32, 33, 34, 35, 36, 37, 38, 39, 40, 41, 42, 43, 44, 45, 46, 47, 48, 49, 50, 51, 52, 53, 54, 55, 56, 57, 58, 59, 60, 61, 62, 63, 64, 65, 66, 67, 68, 69, 70, 71, 72, 73, 74, 75, 76, 77, 78, 79, 80, 81, 82, 83, 84, 85, 86, 87, 88, 89, 90, 91, 92, 93, 94, 95, 96, 97, 98, 99, 100, 102, 103, 104, 105, 106, 107, 108, 109, 110, 111, 112, 113, 114, 115, 116, 117, 118, 119, 120, 121, 122, 123, 124, 125, 126, 127, 128, 129, 130, 131, 132, 133, 134, 135, 136, 137, 138, 139, 140, 141, 142, 143, 144, 145, 146, 147, 148, 149, 150, 151, 152, 153, 154, 155, 156, 157, 158, 159, 160, 161, 162, 163, 164, 165, 166, 167, 168, 169, 170, 171, 172, 173, 174, 175, 176, 177, 179, 180, 181, 182, 183, 184, 185, 186, 187, 188, 189, 190, 191, 192, 193, 194, 195, 196, 197, 198, 199, 200, 201, 202, 203, 204, 205, 206, 207, 208, 209, 210, 211, 212, 213, 214, 215, 216, 217, 218, 219, 220, 221, 222, 223 |
| Only descriptive analyses | 4 (1.8%) | 20, 29, 101, 178 |

**SUPPLEMENTARY MATERIAL 3**

Sample and data characteristics of the papers included in the review with the respective reference number (N=223)

| **Paper characteristics** | **N° papers (%)** | ***References (n° paper)*** |
| --- | --- | --- |
| *Sample Size* | | |
| N<500 | 102 (45.7%) | 1, 2, 5, 6, 11, 15, 16, 17, 18, 19, 20, 21, 22, 27, 30, 31, 34, 37, 39, 42, 44, 46, 47, 48, 49, 50, 53, 54, 56, 58, 59, 60, 61, 65, 71, 72, 75, 76, 77, 78, 82, 85, 86, 88, 90, 93, 95, 96, 101, 102, 103, 108, 111, 112, 113, 114, 69, 118, 119, 120, 121, 122, 126, 130, 134, 137, 139, 144, 146, 147, 148, 154, 155, 156, 157, 158, 162, 163, 164, 165, 172, 173, 175, 176, 178, 179, 181, 185, 191, 192, 193, 197, 201, 202, 203, 204, 205, 208, 217, 218, 220, 223 |
| 501<N<1000 | 60 (26.9%) | 3, 12, 14, 23, 25, 26, 33, 52, 55, 57, 62, 64, 66, 67, 70, 74, 79, 80, 87, 94, 97, 98, 100, 107, 115, 116, 117, 124, 127, 128, 129, 131, 133, 136, 140, 142, 143, 149, 151, 168, 171, 174, 177, 182, 183, 187, 189, 190, 194, 195, 196, 198, 209, 211, 212, 213, 215, 216, 219, 222 |
| N>1000 | 61(27.4%) | 4, 7, 8, 9, 10, 13, 24, 28, 29, 32, 35, 36, 38, 40, 41, 43, 45, 51, 63, 68, 73, 81, 83, 84, 89, 91, 92, 99, 104, 105, 106, 109, 110, 123, 125, 132, 135, 138, 141, 145, 150, 152, 153, 159, 160, 161, 166, 167, 169, 170, 180, 184, 186, 188, 199, 200, 206, 207, 210, 214, 221 |
| *Gender Distribution* | | |
| Balanced | 76 (34.1%) | 1, 3, 4, 9, 10, 14, 20, 25, 27, 29, 31, 32, 33, 35, 37, 39, 41, 45, 46, 49, 50, 51, 56, 58, 60, 61, 66, 73, 75, 76, 77, 79, 80, 81, 82, 86, 88, 91, 92, 93, 96, 97, 100, 103, 104, 108, 112, 115, 117, 119, 120, 121, 124, 125, 130, 131, 132, 136, 137, 140, 145, 148, 151, 153, 154, 160, 162, 181, 184, 187, 196, 200, 208, 210, 217, 223 |
| Female prevalence (over 65%) | 128 (57.4%) | 2, 5, 6, 7, 8, 11, 12, 13, 15, 17, 18, 21, 22, 23, 24, 26, 28, 30, 34, 36, 38, 40, 42, 43, 44, 47, 52, 53, 54, 55, 57, 59, 62, 63, 64, 67, 68, 71, 72, 74, 78, 83, 84, 85, 87, 89, 90, 94, 95, 98, 101, 102, 105, 106, 107, 109, 110, 113, 114, 116, 118, 122, 123, 126, 127, 128, 129, 133, 134, 135, 139, 141, 142, 144, 147, 150, 155, 156, 157, 158, 159, 161, 163, 164, 165, 166, 167, 168, 169, 170, 171, 172, 173, 174, 175, 177, 180, 182, 183, 185, 188, 189, 190, 191, 192, 193, 194, 195, 197, 198, 199, 201, 202, 203, 204, 205, 206, 209, 211, 212, 213, 214, 215, 216, 218, 219, 220, 222 |
| Male prevalence | 5 (2.2%) | 48, 65, 99, 143, 152 |
| Missing | 14 (6.3%) | 16, 19, 69, 70, 111, 138, 146, 149, 176, 178, 179, 186, 207, 221 |
| *Field of study* |  |  |
| Specific area | 73 (32.8%) | 2, 6, 11, 15, 16, 17, 18, 19, 25, 28, 34, 38, 44, 48, 50, 55, 60, 61, 64, 65, 67, 70, 71, 72, 74, 86, 88, 95, 96, 99, 100, 108, 111, 113, 122, 125, 132, 136, 137, 138, 142, 143, 153, 157, 162, 164, 165, 168, 172, 173, 174, 175, 176, 178, 179, 183, 191, 192, 193, 194, 195, 196, 197, 198, 199, 200, 203, 208, 211, 212, 213, 219, 220 |
| Various areas | 75 (33.6%) | 7, 8, 9, 10, 13, 14, 20, 26, 27, 30, 31, 36, 39, 41, 45, 47, 49, 51, 54, 56, 62, 63, 66, 68, 69, 75, 80, 84, 89, 90, 94, 101, 105, 106, 107, 109, 110, 112, 115, 116, 117, 118, 119, 120, 127, 128, 129, 131, 133, 134, 141, 147, 148, 149, 150, 151, 152, 154, 159, 160, 166, 170, 171, 177, 181, 182, 185, 187, 188, 189, 190, 201, 202, 215, 221 |
| Missing | 75 (33.6%) | 1, 3, 4, 5, 12, 21, 22, 23, 24, 29, 32, 33, 35, 37, 40, 42, 43, 46, 52, 53, 57, 58, 59, 73, 76, 77, 78, 79, 81, 82, 83, 85, 87, 91, 92, 93, 97, 98, 102, 103, 104, 114, 121, 123, 124, 126, 130, 135, 139, 140, 144, 145, 146, 155, 156, 158, 161, 163, 167, 169, 180, 184, 186, 204, 205, 206, 207, 209, 210, 214, 216, 217, 218, 222, 223 |
| *Country* | | |
| Italy | 199 (89,2%) | 2, 3, 5, 6, 8, 9, 11, 12, 13, 14, 15, 16, 17, 18, 19, 20, 21, 22, 23, 24, 25, 26, 27, 28, 29, 30, 31, 32, 33, 34, 36, 37, 38, 39, 40, 41, 42, 43, 44, 45, 46, 47, 48, 49, 50, 52, 53, 54, 57, 58, 59, 60, 61, 62, 63, 64, 65, 66, 67, 68, 69, 70, 71, 72, 73, 74, 75, 76, 77, 78, 79, 80, 81, 82, 83, 85, 86, 87, 88, 89, 90, 93, 94, 95, 96, 97, 98, 99, 100, 101, 102, 103, 104, 105, 106, 108, 109, 110, 111, 112, 113, 114, 115, 69, 117, 118, 119, 120, 121, 122, 123, 124, 125, 126, 127, 128, 129, 132, 133, 134, 135, 136, 137, 138, 139, 141, 142, 143, 144, 145, 146, 147, 148, 149, 150, 151, 152, 153, 154, 155, 156, 157, 158, 159, 161, 162, 163, 164, 165, 166, 167, 168, 169, 170, 171, 172, 173, 174, 175, 176, 177, 178, 179, 180, 181, 182, 184, 185, 186, 187, 188, 189, 190, 191, 192, 193, 194, 195, 196, 197, 198, 201, 202, 203, 204, 205, 207, 208, 209, 211, 212, 213, 215, 216, 217, 218, 220, 221, 222, 223 |
| Italy + others | 24 (10.8%) | 1, 4, 7, 10, 35, 51, 55, 56, 84, 91, 92, 107, 116, 130, 131, 140, 160, 183, 199, 200, 206, 210, 214, 219 |
| *Data collection period* | | |
| Pre-COVID-19 | 109 (48.9%) | 2, 3, 6, 8, 9, 10, 11, 12, 14, 15, 18, 21, 22, 23, 25, 27, 29, 30, 32, 34, 35, 37, 38, 39, 40, 41, 42, 46, 48, 49, 50, 51, 52, 54, 57, 58, 60, 66, 72, 73, 75, 79, 80, 82, 84, 87, 91, 92, 95, 96, 98, 99, 100, 102, 104, 113, 115, 116, 124, 125, 130, 132, 133, 135, 137, 139, 142, 145, 149, 150, 151, 152, 153, 154, 157, 161, 162, 163, 164, 166, 167, 168, 171, 172, 173, 175, 176, 179, 181, 182, 183, 185, 187, 188, 189, 190, 191, 192, 193, 196, 200, 203, 205, 207, 208, 210, 213, 219, 221 |
| During COVID-19 (mar 2020-dec 2022) | 80 (35.9%) | 4, 7, 13, 17, 20, 24, 26, 33, 36, 40, 43, 44, 47, 53, 55, 56, 59, 61, 62, 63, 64, 65, 67, 68, 70, 74, 76, 77, 78, 85, 89, 94, 97, 99, 101, 105, 107, 108, 109, 110, 112, 114, 118, 121, 122, 123, 126, 131, 136, 138, 141, 143, 147, 156, 158, 159, 165, 169, 170, 174, 177, 184, 186, 194, 195, 197, 198, 199, 201, 202, 204, 206, 209, 211, 214, 216, 218, 220, 222, 223 |
| Post-COVID-19 | 12 (5.4%) | 5, 28, 69, 71, 90, 106, 119, 120, 127, 128, 129, 215 |
| Missing | 22 (9.8%) | 1, 16, 19, 31, 45, 81, 83, 86, 88, 103, 111, 117, 134, 144, 146, 148, 155, 160, 178, 180, 212, 217 |
